# Contrasting soil seed bank and vegetation dynamics between protected and disturbed arid rangelands in Southern Tunisia

**DOI:** 10.3389/fpls.2026.1743624

**Published:** 2026-04-24

**Authors:** Abderrazak Tlili, Mohamed Neffati, Kamel Dadi, Azaiez Ouled Belgacem

**Affiliations:** 1 Animal and Forage Productions Laboratory (LR16 INRAT 01), National Institute of Agronomic Research of Tunisia (INRAT), Carthage University, Tunis, Tunisia; 2 Laboratory of pastoral ecosystem and valorization of spontaneous plants and associated microorganisms, Institut des Régions Arides Médeninee (IRA), University of Gabès, Médenine, Tunisia; 3 International Center for Agricultural Research in the Dry Areas, Tunis, Tunisia; 4 Laboratory of Eremology and Combating Desertification, Institut des Régions Arides Médenine (IRA), Médenine, Tunisia

**Keywords:** arid rangelands, ecosystem resilience, grazing management, protected areas, soil seed bank

## Abstract

**Introduction:**

Over recent decades, accelerated degradation of arid rangelands has led to significant losses in ecosystem function, in some cases surpassing irreversible thresholds that hinder restoration. Soil seed banks (SSBs) serve as critical ecological reservoirs influencing vegetation succession and ecosystem resilience after disturbance.

**Methods:**

This study investigates the effects of grazing on both above-ground vegetation (AGV) and SSB dynamics. It evaluates the efficacy of long-term protection as a restoration strategy in arid ecosystems. We compared a protected area (a national park) with adjacent continuously grazed rangelands by monitoring above-ground vegetation during the 2022 spring growing season and conducting an emergence test on soil samples collected in summer 2022 at two depths (0–3 cm and 3–6 cm).

**Results and discussion:**

Protection substantially increased vegetation cover and improved surface properties, including litter accumulation and the development of biological soil crusts. The protected site supported persistent perennial, medium- to high-palatability species, while grazed areas exhibited higher SSB density and richness dominated by ephemeral, disturbance-tolerant taxa associated with sparse vegetation and highly disturbed soils. Sorensen’s similarity coefficient (SSCI) indicated high similarity between above-ground vegetation inside versus outside protection (0.77), moderate similarity between SSBs (0.62), and a greater decoupling between above- and below-ground components in the protected area (0.43) compared to grazed rangelands (0.57). Despite the similarity in shared species occurring under both management regimes, total vegetation cover, plant density, and species richness increased significantly under protection, highlighting the negative impact of continuous grazing in arid environments. However, when combined with aridity, plant senescence and surface crust formation may create barriers to regeneration and limit the long-term dynamics of both AGV and the SSB under prolonged protection. These findings indicate that both prolonged protection and continuous grazing can diminish regeneration potential and reduce resilience in arid rangelands. Accordingly, controlled grazing regimes that balance disturbance with sufficient recovery periods, coupled with regular monitoring of SSB, are critical for maintaining ecosystem function and resilience. Further research is needed to investigate SSB dynamics under different management practices, particularly at sites where controlled grazing systems are implemented.

## 1 Introduction

Arid rangelands provide critical ecosystem services, including biodiversity conservation and livestock grazing (Gamoun et al., 2018), yet they are highly susceptible to degradation. Mismanagement and the shift from traditional controlled grazing to continuous, unrestricted grazing have reduced productivity, forage quality, and biodiversity, often favoring unpalatable species over highly palatable ones (Nefzaoui et al., 2011; Ouled Belgacem et al., 2013a, Ouled Belgacem et al., 2019; Louhaichi et al., 2021; Ould Sidi Mohamed et al., 2002; Jauffret and Visser, 2003; Tarhouni et al., 2010; Palacio et al., 2014).

The soil seed bank (SSB) is a vital component of ecosystem resilience and vegetation regeneration, serving as an on-site source of viable seeds that support plant diversity and ecological succession (Wellstein et al., 2007; Chu et al., 2019; Miaojun et al., 2018; Hu et al., 2019). Despite its importance, few studies have characterized SSBs at the ecosystem level, particularly in Tunisian arid rangelands.

The SSB reflects both current and historical vegetation and provides a latent stage in the plant life cycle, offering insights into future community structure (Kalamees et al., 2012; Hu et al., 2019). Seeds may be autochthonous or allochthonous, and exhibit either transient (short-lived, non-dormant) or persistent (dormant, long-lived) behaviors (Dos Santos et al., 2018).

SSB composition is influenced by environmental factors—such as precipitation, temperature, light, and humidity—as well as biotic and anthropogenic pressures, including predation, pathogens, and disturbances (Dos Santos et al., 2013, Dos Santos et al., 2016). Dormancy, which prevents germination under favorable conditions, can be primary or secondary, depending on physiological traits and environmental cues (Benech-Arnold et al., 2000).

Human disturbances influence soil seed banks (SSBs) both directly and indirectly, with agricultural practices, rangeland conversion, and overgrazing among the most reported factors (Wellstein et al., 2007; Dos Santos et al., 2018; Chu et al., 2019). In pastoral ecosystems, livestock grazing, through selective consumption of palatable species, seed dispersal, and alterations of soil properties via trampling and nutrient deposition, affects both above-ground vegetation (AGV) and SSB dynamics (Dong et al., 2012; Chu et al., 2019). Grazing intensity strongly influences SSB composition: light grazing can enhance richness, while moderate to heavy grazing often reduces it, though increased soil disturbance may favor annual species and enlarge the seed bank (Shi et al., 2022; Miaojun et al., 2018). Short-term grazing exclusion generally benefits AGV and SSB, whereas Long-term exclusion can have negative effects by promoting the formation of soil crusts, which can significantly impact the seed bank (Li et al., 2005). Crusted soils often reduce both the percentage and the speed of seed germination compared to uncrusted soils, thereby limiting vegetation cover and seedling establishment in both arid and more mesic ecosystems (Huang et al., 2022; Shi et al., 2022). Continuous grazing can also reduce the compositional similarity between AGV and SSB, potentially disrupting regeneration (Shen et al., 2018). Understanding these interactions is critical for predicting successional trajectories and guiding sustainable management, as SSBs play a pivotal role in vegetation recovery following disturbances such as overgrazing and drought.

In this study, we aim to compare species composition, richness, seed density, soil surface materials, and the degree of similarity between above-ground vegetation (AGV) and the soil seed bank (SSB) in both protected and continuously grazed areas. Specifically, we address the following questions: (1) Why do long-term protection and continuous grazing lead to a weakened SSB? (2) Why do these management regimes increase the contribution of transient annual species? (3) What are the practical implications of these findings for the management of arid ecosystems?

We hypothesize that both long-term protection and continuous grazing exert negative effects on ecosystem dynamics by disrupting community assembly, altering successional trajectories, and impacting ecosystem services. In continuously grazed areas, we expect the soil seed bank (SSB) to be dominated by short-lived annual species, resulting in a process of therophytization in the above-ground vegetation. Conversely, in long-term protected areas, succession is anticipated to favor the accumulation of senescent woody species, reflecting shifts in seed input and storage dynamics due to reduced disturbance. In case of intermediate disturbance, is the protected area becoming senescent or dominated by a few shrub species that do not form persistent seed banks?

## 2 Materials and methods

### 2.1 Study area

**Figure 1:**
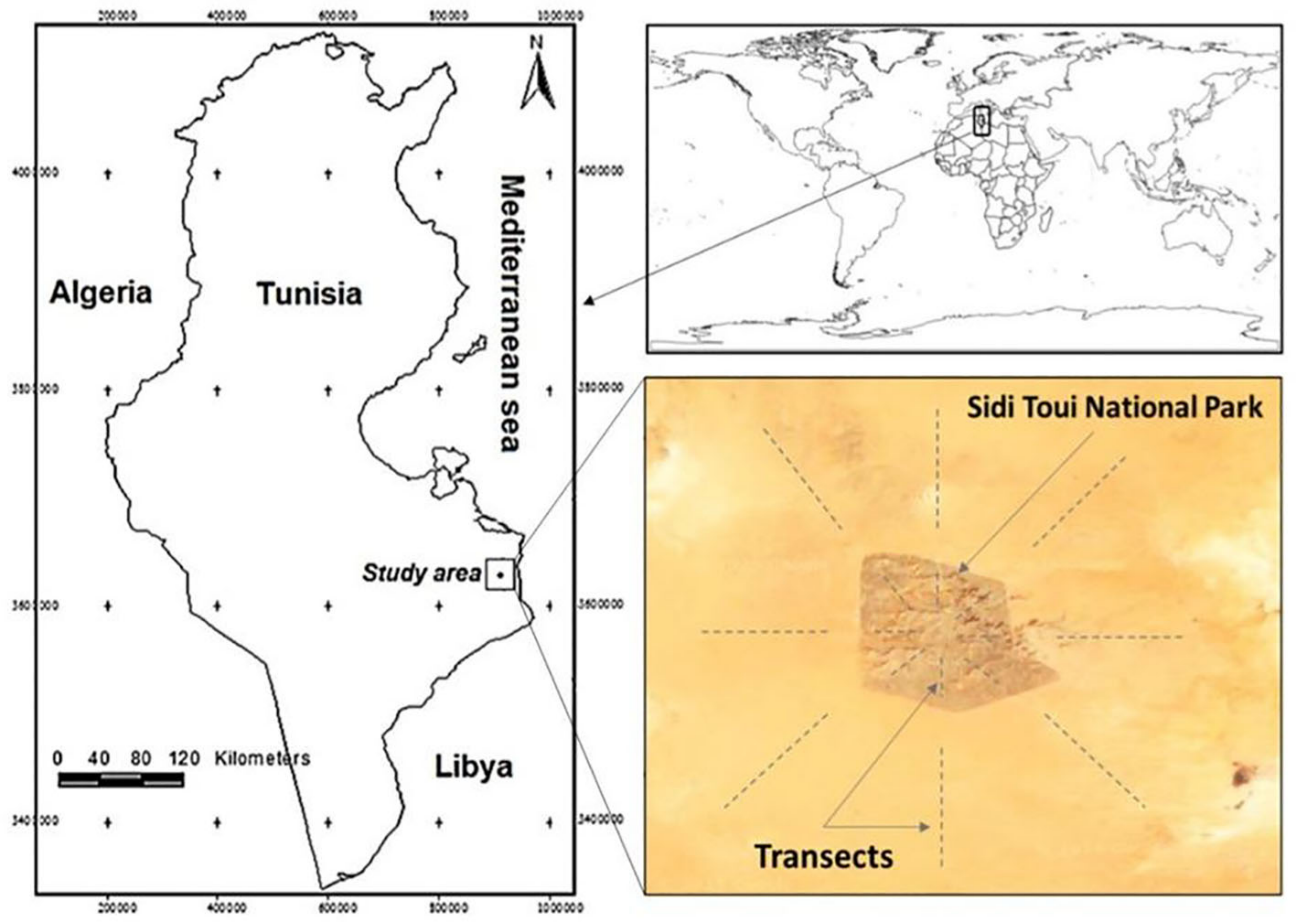
Study area localization. Sidi Toui National Park (protected area) and their surroundings (grazed area). Dashed lines indicate the localization of monitored transects in the protected (inside) and grazed (outside) areas.

The study was conducted in the El Ouara Ben Guerdane natural rangelands, specifically within the Sidi Toui National Park (a protected area) and its surrounding grazed areas, located in southern Tunisia (**Figure 1**; 32°42’45.0”N, 11°14’57.4”E). The soil in this region is primarily composed of encrusted glacis with a sandy and shallow upper layer. Vegetation is mainly characterized by shrubby chamaephytes, grasses, and forbs.

Historically, livestock grazing—mainly by small ruminants and camels—has been the dominant land use in the surrounding free-access rangelands. In contrast, grazing is strictly prohibited within the Sidi Toui National Park, which was established in 1991 and covers an area of 6,315 hectares. The park hosts only a small number of wild herbivores, including Oryx, gazelles, as well as ostriches and rabbits.

The region experiences a hot desert climate (BWh, Köppen-Geiger classification), with a mean annual temperature of 20.6 °C and mean annual precipitation of approximately 195 mm (estimated data from 1991 to 2021). July is the driest month, while the highest rainfall typically occurs in December (https://fr.climate-data.org, accessed on 9 August 2025). Monthly rainfall measurements were taken in site during the years 2019, 2020, 2021, and 2022, indicating a prolonged and extremely dry period, and are presented in **Table 1**. In recent decades, significant climate changes have caused scarce and irregular rainfall, leading to disruptions in both the seasonal distribution and the number of rainy days. During autumn 2021, a cumulative rainfall of 30 mm was recorded in October, following an annual rainfall of approximately 114.5 mm in the previous year (2020–2021) within the study area. This level of autumn rainfall was sufficient to trigger the germination of most plant seeds in arid regions, confirming the suitability of this period for conducting the study.

**Table 1:** Annual rainfall recorded in the study area during the period from September 2019 to August 2022.

Year	Sep	Oct	Nov	Dec	Jan	Feb	Mar	Apr	May	Jun	Jul	Aug	Annual rainfall (mm/year)
19-20	0	0	0	0	0	0	14	4	0	0	0	0	18
20-21	0	0	17	107.5	0	0	0	0	0	0	0	0	114.5
21-22	0	30	0	0	0	0	0	0	0	0	0	0	30

Data source: Commissariat Régional au Développement Agricole de Médenine (accessed on November 5^th^, 2025).

### 2.2 Experimental design

Two areas were distinguished according to the management mode: (i) Protected area (inside the park) and (ii) Grazed area (the surroundings of the park). Starting from the middle of the park, and for each of the eight directions (North “N”, Northeast “NE”, East “E”, Southeast “SE”, South “S”, Southwest “SW”, West “W” and Northwest “NW”), linear transects were randomly designated (8 inside and 8 outside). In each direction, three plots inside the park and five outside were randomly selected to have a total of 24 and 40 plots in the protected and grazed areas, respectively. The five plots in the grazed area, for each direction, are randomly distributed on a straight line perpendicular to the borderline of the protected area to avoid the proximity effect. At each plot, AGV and soil surface materials monitoring were carried out in spring (late February-March 2022, a peak of the vegetative growth period). Soil sampling was conducted across all plots in August 2022, during the summer season, targeting two depth intervals (0–3 cm and 3–6 cm). Samples were collected randomly from inter-patch zones (bare soil between vegetation tufts). The soil samples were subsequently tested for germination under controlled conditions designed to simulate natural environments.

### 2.3 Vegetation and soil surface materials monitoring

In each plot, a comprehensive floristic inventory was carried out, documenting the floristic composition and highlighting the dominant species recorded during the survey. Vegetation cover and soil surface materials were assessed using the quadrat point method, which involves systematically recording observations at fixed points within a defined sampling area (Daget and Poissonnet, 1971) in both protected and grazed areas during the spring of 2022. In each plot, a randomly placed 20-meter line transect was established. Along each line transect, a fine pin was vertically lowered at 20 cm intervals, recording contacts with vegetation, and the interceptions between the vegetation and ground surface components (bare-soil, soil-crust, eolian veil, gypsum crust, litter, and stones). Additionally, three subplots were established at each plot, including one large subplot (20 m^2^) for measuring the perennial plant density, within which two small subplots (1 m^2^ each) were used to assess annual species. Vegetation cover (VC) was calculated as:


$$\text{VC}=(\frac{n}{N})\text{x}\;100$$

Where *n* represents the number of points with vegetation presence and *N* represents the total number of points. To compare the plant diversity between the protected and grazed areas, the Sørensen coefficient similarity index was calculated as:


VC=2j/(a+b)

Where *a* and *b* are the number of species in the soil seed bank and AGV, respectively, and *j* is the number of species shared (Shang et al., 2016). Specific frequency (F) was calculated as:


F=(nplots/Nplots) x 100

Where *nplots* represents the number of sampling plots where the species occurs, and *Nplots* represents the total number of sampling plots studied.

For unidentified species, samples were collected and identified using botanical guides and specific databases.

### 2.4 Soil sampling

Soil sampling was conducted in summer (August 2022), after seed production and dispersal had occurred for most plant species in the study area. At each plot, three soil cores were collected, and samples were taken from two depths: 0–3 cm and 3–6 cm (following Guo et al., 1998; Snyman, 2004), as approximately 97% of seeds are typically found within the top 0–5 cm of soil. Samples from the same depth within each plot were pooled and thoroughly mixed to ensure homogeneous seed distribution. In total, 128 soil samples were obtained (24 and 40 plots × 2 depths from protected and grazed areas, respectively). The samples were then placed outdoors for six months (autumn and winter) to expose the seeds to low temperatures (vernalization), after which they were transferred to a controlled greenhouse for germination assays.

### 2.5 Emergence test

Large litter fragments and stones were removed from the soil samples. The purified samples were then mixed to ensure homogeneity and preserve the integrity of the seeds. Each soil sample (3 cm thick) was placed on a 3 cm layer of inert, seed-free sand in a germination tray (15 × 20 cm). The trays were placed outdoors for six months during autumn and winter to expose the seeds to low temperatures (vernalization). After this period, they were transferred to a controlled greenhouse for germination assays. Germination trays were maintained under controlled greenhouse conditions with consistent moisture, a temperature of 20 ± 5 °C, relative humidity between 65% and 85%, and a photosynthetic photon flux density (PPFD) of approximately 1,200 µmol m^-2^ s^-1^, under a 10/14 h light/dark photoperiod. During the initial three months, all emerged seedlings were identified and removed, while those that could not be identified were retained until identification was possible. Following a second vernalization cycle and subsequent cessation of irrigation, the germination test was repeated for an additional three months using the same trays. The total number of emerging seedlings across both cycles was used to estimate the number of viable seeds and to characterize the soil seed bank composition. Non-viable seeds that remained ungerminated were not included in the analysis.

### 2.6 Data analysis

To analyze grazing effects, species richness and density (annuals, perennials, and totals) were recorded. The Sørensen similarity index was applied to assess similarities in species composition between the soil seed bank and aboveground vegetation (AGV), both between and within grazed and protected areas. Normality testing using the Shapiro-Wilk test indicated that the data were non-normally distributed, except for total richness. Consequently, Kruskal-Wallis ANOVA was used to compare vegetation cover, soil surface material proportions, annual and perennial densities, and richness. Mean comparisons were performed using Dunn’s test at a significance level of p < 0.05. Redundancy Analysis (RDA) was performed to visualize the impact of grazing and protection on plant communities. This analysis used a combination of explanatory variables—life cycle, palatability index (Le Houérou and Ionesco, 1987), and germination area—to explain the variation in germinated species frequency in both the aboveground vegetation and soil seed bank. All statistical analyses were carried out using R version 4.5.1, except for RDA, which we used OriginPro 2025b software.

## 3 Results

### 3.1 Vegetation cover and soil surface features

**Figure 2:**
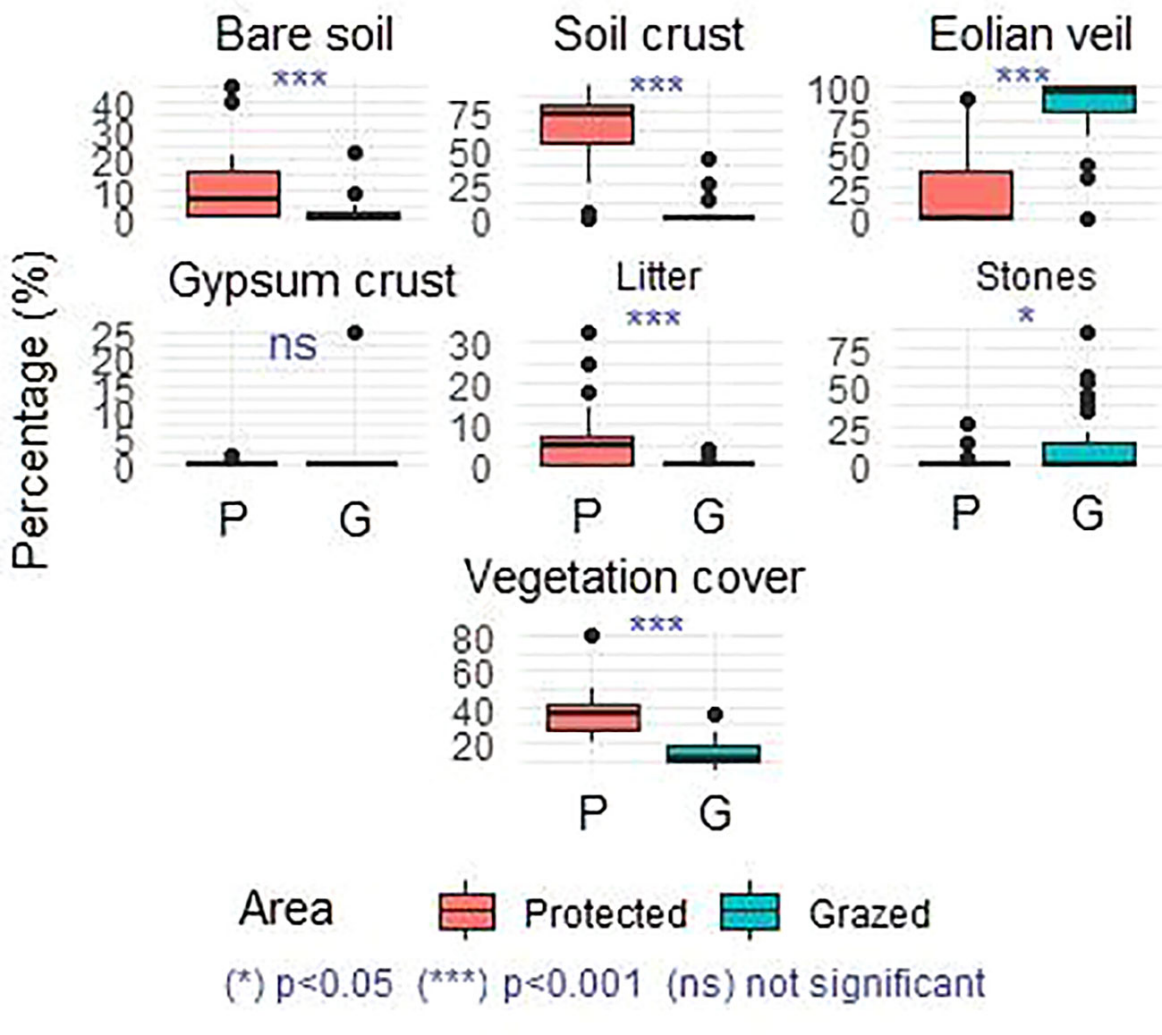
Soil surface materials and Vegetation cover (%) in the monitored plots during spring of 2022. P, protected area (Sidi Toui National Park); G, Grazed area (surroundings of the park). Asterisks indicate a significant difference (p<0.05) and “ns” indicates a non-significant difference (p>0.05).

The analysis of vegetation cover (VC) and soil surface characteristics revealed that, aside from plant tufts, five dominant surface features were identified: bare soil, soil crust, eolian veil, litter, and stones. Variations in VC and the relative proportions of these soil features within protected and grazed areas are presented in **Figure 2**.

A non-parametric analysis of variance (Kruskal–Wallis test), followed by Dunn’s *post-hoc* test, indicated highly significant differences (p < 0.001) for VC as well as for all soil surface components. The highest VC values were recorded inside the protected area, with a significant decrease observed in the grazed area.

Within the protected area, the mean proportions of soil crust, litter, and bare soil increased significantly, whereas eolian veils and stones decreased. This pattern suggests a positive correlation between VC, litter accumulation, bare soil, and soil crust development, likely resulting from moderate herbivore activity (light grazing and trampling), which enhances vegetative growth, aerial phytomass accumulation, and surface crust formation.

Conversely, in the grazed area, a clear dominance of eolian veil and stones was observed, reflecting intense disturbance primarily due to grazing. This high level of pressure accelerates soil degradation processes, highlighting the negative impact of overgrazing on surface stability and vegetation cover.

### 3.2 Aboveground species composition, density and richness

**Figure 3:**
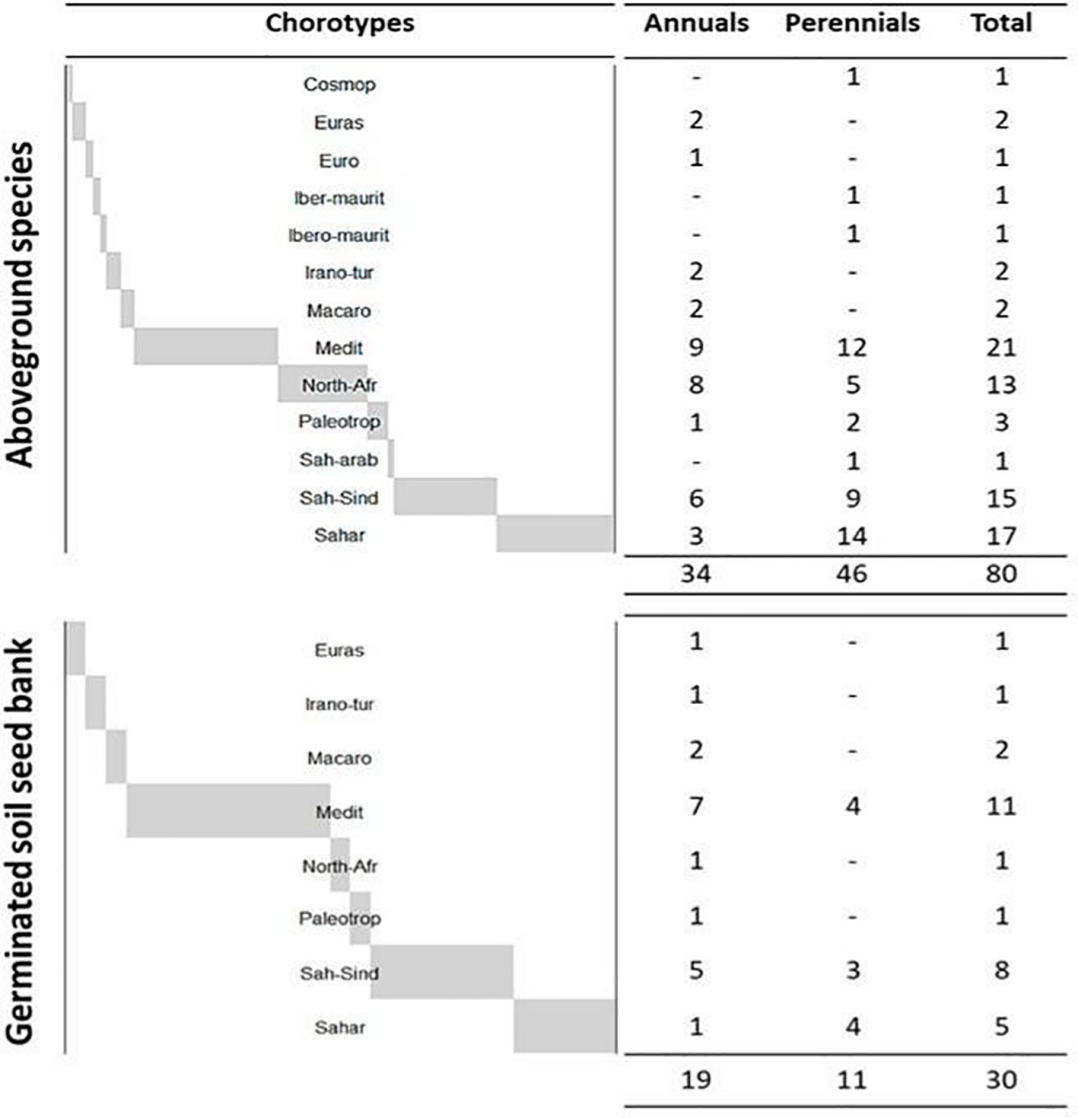
Floristic composition of the study area: Aboveground species and germinated soil seed bank species richness according to life cycle (annuals vs. perennials) and chorotypes.

**Figure 4:**
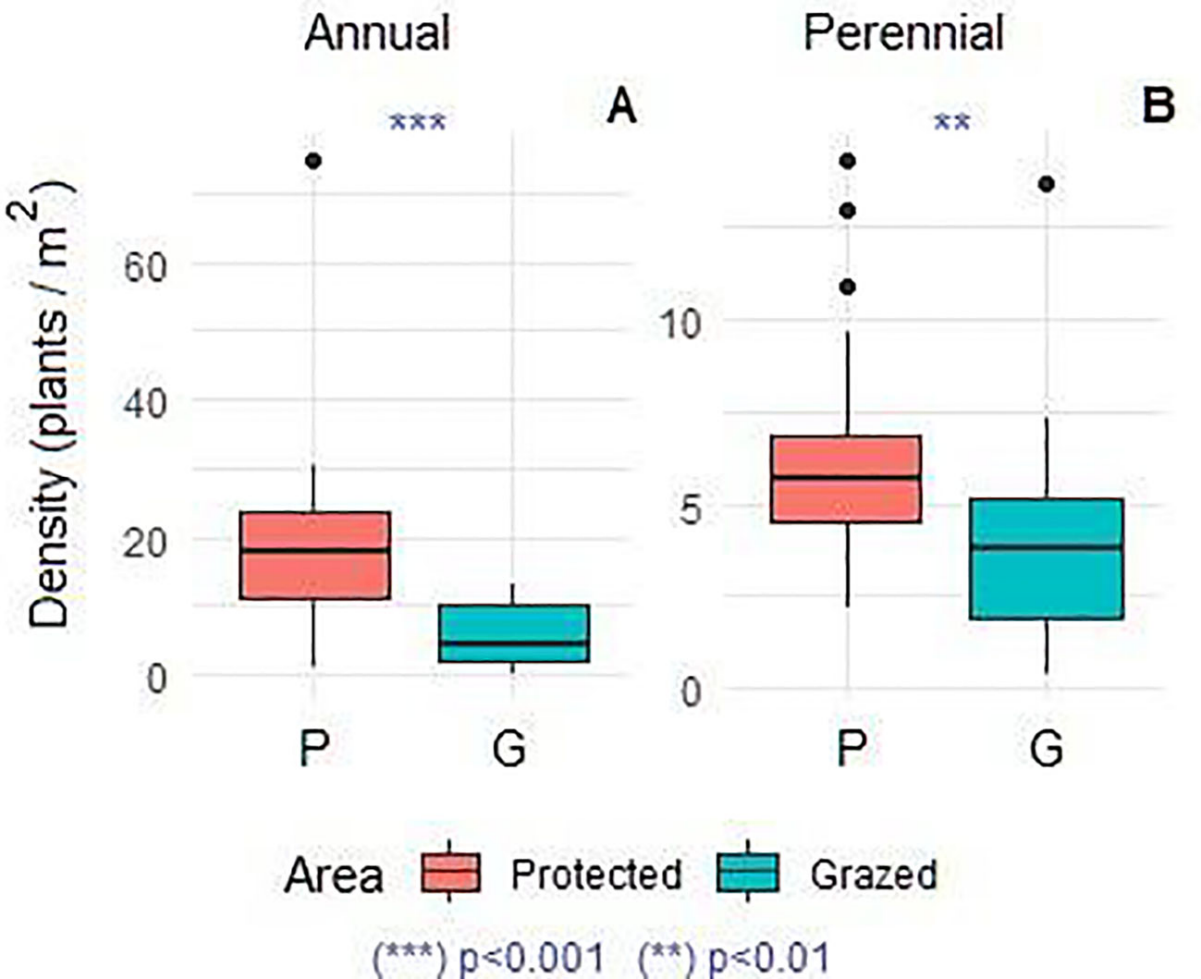
Comparison of aboveground annual **(A)** and perennial density **(B)** in protected and grazed areas. Kruskal-Wallis (Dunn’s test). N, 24 protected plots and 40 grazed plots. Asterisks indicate a significant differences (p<0.01 and p<0.001).

**Figure 5:**
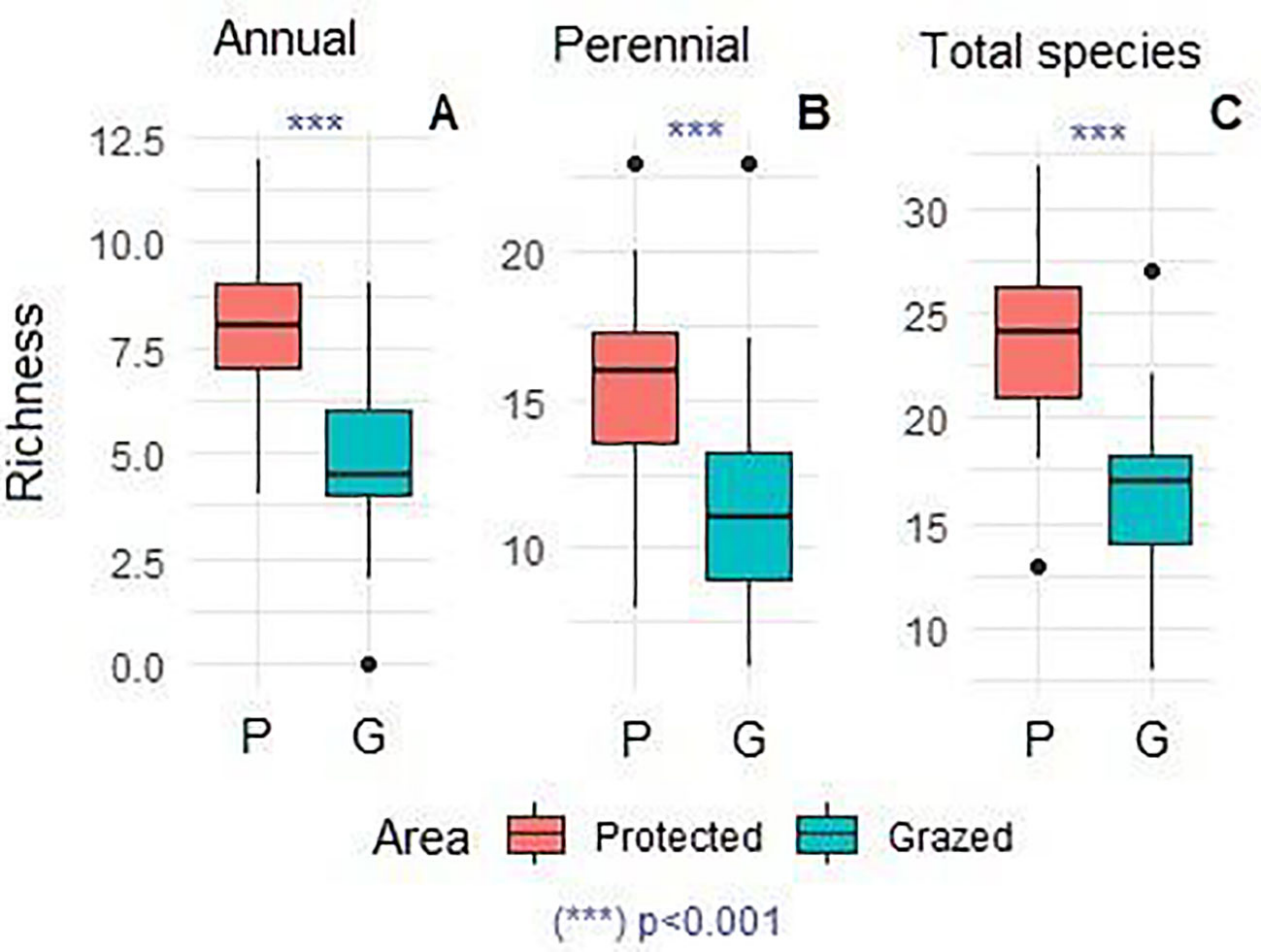
Comparison of aboveground annual richness **(A)**, perennial richness **(B)**, and total richness **(C)** in both protected and grazed areas. Kruskal-Wallis (Dunn’s test). N, 24 protected plots, and 40 grazed plots. Asterisks indicate a highly significant difference (p<0.001).

The floristic composition recorded in the study area comprises a total of 80 species, including 46 perennials and 34 annuals (**Figure 3**). Most represented families were Asteraceae (16 species; 20%), Poaceae (13 species; 16.25%), Fabaceae (10 species; 12.5%), Amaranthaceae (6 species; 7.5%), Brassicaceae (5 species; 6.25%), Caryophyllaceae and Plantaginaceae (with 4 species for each family; 5%) and other families less presented comprising together 22 species (27.5%). The remaining families each contributed one species (3%). The dominant chorotypes are primarily Mediterranean, Saharan, Saharo-Sindian, and North-African, with fewer Paleotropical elements, represented by 21 (24%), 17 (21%), 15 (20%), 13 (16%), and 3 (4%) species, respectively. A comparison of the mean densities of annual and perennial species is presented in **Figure 4**. Analysis of variance revealed a highly significant increase in the density of annual species (p < 0.0001) and a significant increase in perennial species (p < 0.01) in the protected area compared to the grazed area. Similarly, annual, perennial, and total species richness increased significantly (p < 0.001), demonstrating the positive effect of grazing exclusion (**Figure 5**).

Examination of species similarity between the protected and grazed areas showed a high Sorensen’s Coefficient of Similarity Index (SCSI = 0.77), with 50 species shared between the two sites out of 73 species recorded in the protected area and 57 in the grazed area (**Table 2**). This index indicates that although the floristic compositions differ, the two areas share a substantial number of species. This similarity may be explained by the presence of species that are probably well adapted to grazing pressure.

**Table 2:** Sørensen coefficient similarity index (SCSI) between aboveground species in protected and grazed area.

Paired area	Species presence		SCSI
	Present species	Shared species	
Protected	73	50	0.77
Grazed	57		

### 3.3 Density of germinated soil seed bank

**Figure 6:**
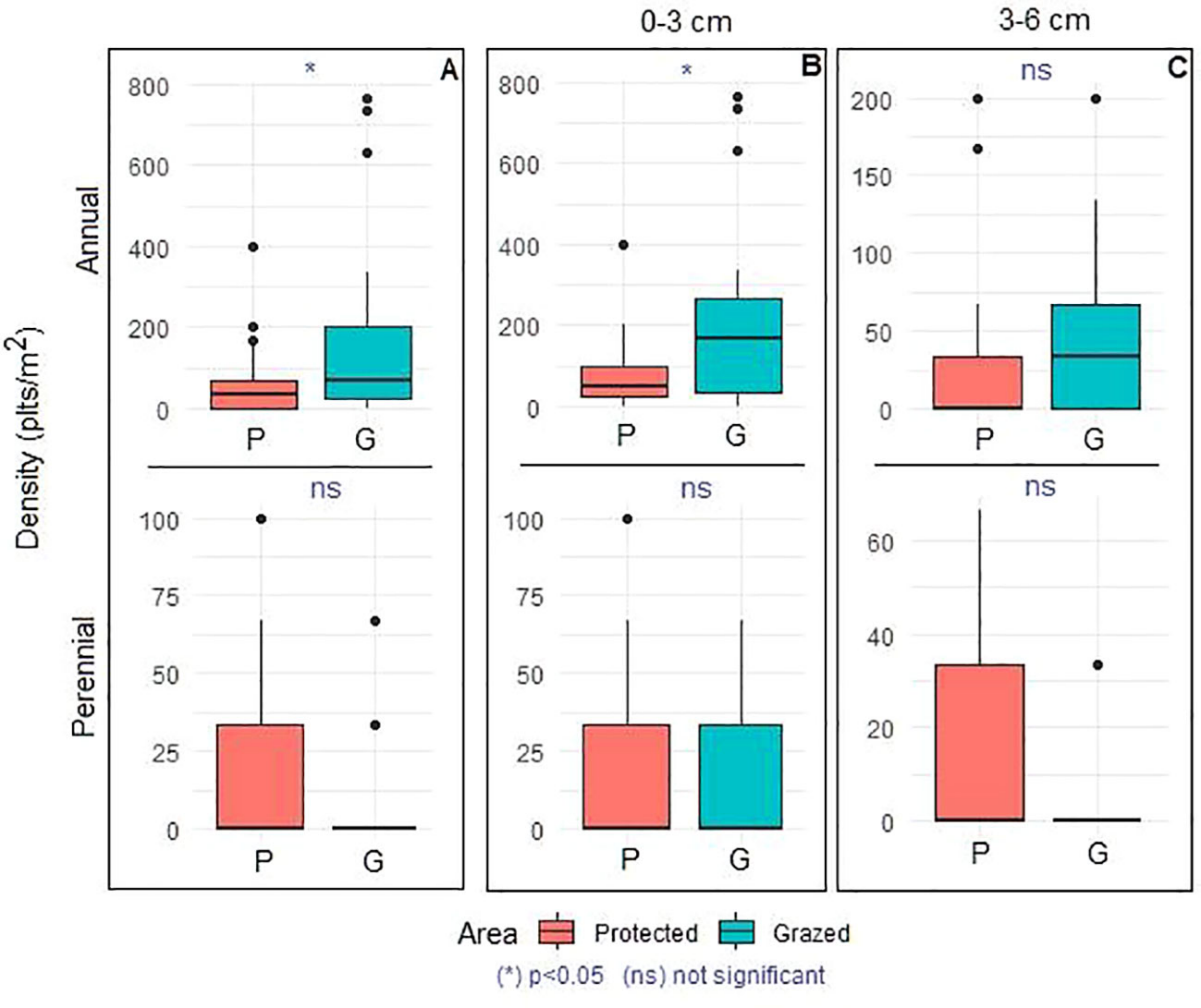
Comparison of annual and perennial densities of germinated soil seed bank **(A)** according to grazing (protected and grazed area) and **(B, C)** according to soil depth between and within protected and grazed areas, (n = 24). Kruskal-Wallis (Dunn’s test). Asterisks indicate a significant differences (p<0.05) and “ns” indicates a non-significant difference (p>0.05).

A total of 30 plant species were recorded in the germinated soil seed bank, comprising 11 perennial and 19 annual species (**Figure 3**). The most dominant chorotypes were Mediterranean, Saharo-Sindian, and Saharan, represented by 11 (36.66%), 8 (26.66%), and 5 (16.66%) species, respectively. The variation in the density of annual and perennial species germinated from the soil seed bank is illustrated in **Figure 6**. A comparison of mean densities showed that annual species were significantly more abundant in the grazed area than in the protected area (*p* < 0.013), whereas no significant difference was observed for perennial species (*p* = 0.36). When mean densities were further compared according to soil depth (0–3 cm and 3–6 cm), annual species exhibited significantly higher emergence in the grazed area at the 0–3 cm depth (*p* < 0.001), while perennial species showed no significant variation with soil depth (*p* = 0.116).

### 3.4 Species richness and composition in soil seed bank

**Figure 7:**
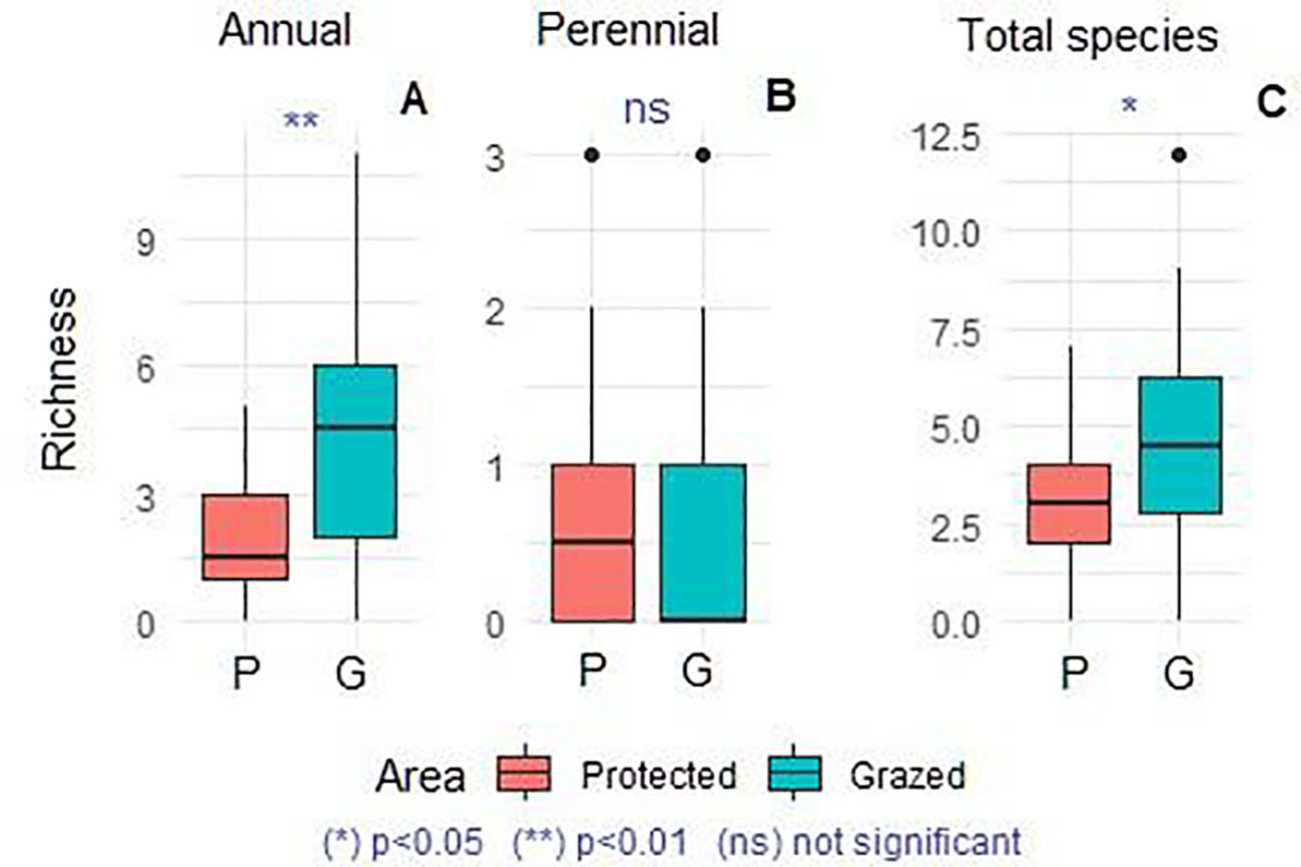
Comparison of annual, perennial **(A, B)** and total richness **(C)** of germinated soil seed bank **(A)** between protected and grazed area, (n = 24). Kruskal-Wallis (Dunn’s test). Asterisks indicate a significant differences (p<0.05) and “ns” indicates a non-significant difference (p>0.05).

**Figure 7** compares the species richness of annuals, perennials, and the total germinated soil seed bank between protected and grazed areas. Mean comparison indicates a highly significant difference in total and annual species richness (p = 0.043; p < 0.01), with the grazed area soils exhibiting higher mean values. However, no significant difference was observed in perennial species richness. More than two-thirds of the germinated species were annual, representing 14 botanical families (**Table 3**). The most abundant families were Fabaceae (7 species; 23%), Asteraceae (5 species; 17%), Poaceae (4 species; 13%), and Plantaginaceae (3 species; 10%). The remaining families each contributed one species (3%). Comparing these proportions with the AGV, we note a similar dominance of Asteraceae, Poaceae and Fabaceae, an increased presence of Plantaginaceae with a notable decrease of Amaranthaceae, Brassicaceae and Caryophyllaceae in SSB. Based on the indices of Le Houérou and Ionesco (1987), 30% of the emerged species were highly palatable, 37% moderately palatable, 17% rarely palatable, and only 6% unpalatable.

**Table 3:** Germinated soil seed bank species in protected vs. grazed areas: Presence (+)/absence (-), life cycle (P: perennial, A: annual), and palatability index (Le Houérou and Ionesco, 1987).

N°	Species	Life cycle	Palatability index	Emergence (P/A)	
				Protected plots	Grazed plots
1	*Anabasis articulata*	P	1	–	+
2	*Argyrolobium uniflorum*	P	5	–	+
3	*Asphodelus tenuifolius*	A	0	+	+
4	*Astragalus corrugatus*	A	2	+	+
5	*Bromus madritensis*	A	2	+	+
6	*Cutandia dichotoma*	A	4	+	+
7	*Daucus sahariensis*	A	2	+	+
8	*Erodium glaucophyllum*	P	1	+	–
9	*Fagonia glutinosa*	P	0	+	+
10	*Filago germanica*	A	1	+	+
11	*Hedysarum spinosissimum*	P	3	+	+
12	*Helianthemum kahiricum*	P	4	+	+
13	*Helianthemum lippii*	P	5	+	+
14	*Hippocrepis bicontorta*	A	4	+	–
15	*Ifloga spicata*	A	1	+	+
16	*Launaea glomerata*	P	4	–	+
17	*Launaea nudicaulis*	P	2	–	+
18	*Lotus halophilus*	A	3	–	+
19	*Matthiola longipetala*	A	2	+	+
20	*Medicago minima*	A	3	–	+
21	*Medicago truncatula*	A	5	+	+
22	*Mollugo cerviana*	A	1	–	+
23	*Neurada procubaines*	A	2	+	–
24	*Paronychia arabica*	A	2	+	+
25	*Plantago albicans*	P	5	+	+
26	*Plantago coronopus*	A	2	–	+
27	*Plantago ovata*	A	3	+	+
28	*Senecio glaucus*	A	1	+	–
29	*Schismus barbatus*	A	4	+	+
30	*Stipa lagascae*	P	4	+	+

A species was considered germinated if it emerged in at least one of the 48 germination trays across the protected and grazed areas (with depth treatments combined).

### 3.5 Similarity indices between the germinated SSB and aboveground species

**Table 4** presents the comparison of floristic composition between the aboveground vegetation (AGV) and the germinated soil seed bank, both within and between the protected and grazed areas. The Species Composition Similarity Index (SCSI) was used to evaluate the degree of similarity in species composition between paired areas—specifically, between AGV and the soil seed bank in each area, and between the germinated soil seed banks of the protected and grazed sites.

**Table 4:** Sørensen coefficient similarity index (SCSI) between aboveground vegetation (AGV) and germinated soil seed bank (SSB) in protected area vs. grazed area and between germinated soil seed bank in the protected vs. those in grazed area.

Comparison’s element	Paired area	Species presence		SCSI
		Present species species	Shared species	
Protected area	AGV	73	20	0.43
	SSB	21		
Grazed area	AGV	57	24	0.57
	SSB	27		
Germinated SSB	Protected	21	17	0.62
	Grazed	27		

A low similarity (SCSI = 0.43) was observed between the AGV and the germinated soil seed bank in the protected area, where 20 species were shared out of 21 emerged seedlings and 73 living plants. In contrast, the grazed area exhibited a higher similarity (SCSI = 0.57), sharing 24 species between 27 emerged seedlings and 57 living plants. When comparing the germinated soil seed banks between the protected and grazed areas, a higher similarity (SCSI = 0.62) was recorded, with 17 species shared between the 21 and 27 germinated species in the protected and grazed areas, respectively.

Overall, the grazed soil seed bank exhibited a greater number of emerged seedlings, indicating a higher germination potential compared to the soil seed bank of the protected area.

### 3.6 Relationship between frequency and traits of germinated species

The frequency of species observed during germination tests and their corresponding occurrence in the aboveground monitored is shown in **Table 5**. The analysis of the AGV species occurrence reveals that the highest frequency was registered in protected plots by *Helianthemuum kahiricum* (100%), *Helianthemum lippii* (88%), *Matthiola longipetala* (83%) and medium frequencies by *Astragalus corrugatus* (67%), *Hippocrepis bicontorta* (63%), *Medicago minima* (63%), *Plantago albicans* (58%), *Plantago ovata* (58%) and *Erodium glaucophyllum* (54%). In the grazed plots, the AGV depicted a high frequency only for *Argyrolobium uniflorum* (100%) followed by moderate occurrences of *Plantago albicans* (67%), *Asphodelus tenuifolius* (67%), *Helianthemum kahiricum* (58%), *Helianthemum lippii* (58%), *Medicago minima* (54%) and *Stipa lagascae* (50%).

**Table 5:** Specific frequency (Percentage of sample plots where a similar species was found) of the germinated soil seed bank (SSB) and aboveground vegetation (AGV) in protected and grazed areas.

N°	Species	Frequency (%)			
		AGV		SSB	
		Protected plots	Grazed plots	Protected plots	Grazed plots
1	*Anabasis articulata*	25	21	0	4
2	*Argyrolobium uniflorum*	58	100	0	21
3	*Asphodelus tenuifolius*	13	67	0	21
4	*Astragalus corrugatus*	67	21	17	4
5	*Bromus madritensis*	0	4	8	17
6	*Cutandia dichotoma*	4	38	4	8
7	*Daucus sahariensis*	8	17	8	4
8	*Erodium glaucophyllum*	54	8	4	0
9	*Fagonia glutinosa*	21	8	8	4
10	*Filago germanica*	0	8	29	46
11	*Hedysarum spinosissimum*	8	0	8	4
12	*Helianthemum kahiricum*	100	58	13	8
13	*Helianthemum lippii*	88	58	17	8
14	*Hippocrepis bicontorta*	63	17	4	0
15	*Ifloga spicata*	8	25	29	58
16	*Launaea glomerata*	0	0	4	4
17	*Launaea nudicaulis*	4	0	0	4
18	*Lotus halophilus*	8	33	0	8
19	*Matthiola longipetala*	83	13	8	8
20	*Medicago minima*	63	54	8	8
21	*Medicago truncatula*	0	4	0	38
22	*Mollugo cerviana*	0	4	0	4
23	*Neurada procumbens*	8	25	8	0
24	*Paronychia Arabica*	21	8	17	38
25	*Plantago albicans*	58	67	17	4
26	*Plantago coronopus*	4	0	0	4
27	*Plantago ovate*	58	13	17	17
28	*Senecio glaucus*	17	21	13	50
29	*Schismus barbatus*	8	0	4	0
30	*Stipa lagascae*	33	50	4	4

Conversely, grazed plot soils showed higher seedling frequencies with *Ifloga spicata* (58%), *Schismus barbatus* (50%), *Filago germanica* (46%), *Paronychia arabica* (38%) and *Medicago truncatula* (38%). The protected plot soils exhibited lower frequencies, represented mainly by *Filago germanica* (29%), *Ifloga spicata* (29%), *Paronychia arabica* (17%), *Plantago ovata* (17%), *Astragalus corrugatus* (17%), *Helianthemum lippii* (17%) and lowest frequencies recorded for *Helianthemum kahiricum* (13%) and *Schismus barbatus* (13%).

**Figure 8:**
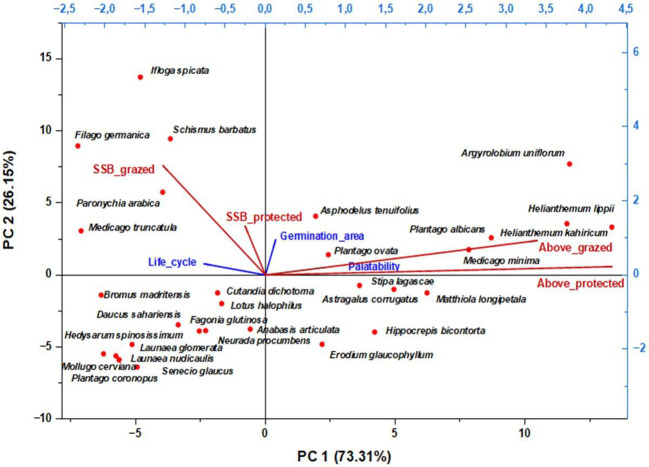
Redundancy analysis (RDA) of aboveground vegetation and soil seed bank in protected vs. grazed Areas: a biplot highlighting key relationships with life cycle, germination area, and palatability Traits. Response variables: SSB_protected = frequency in soil seed bank protected area, SSB_grazed = frequency in soil seed bank in grazed area, Above_protected = frequency in aboveground protected area, Above_grazed = frequency in aboveground grazed area.

To assess the effect of the management mode on plant cover dynamics, we compared aboveground (living plants) and underground (germinated soil seed bank) species groups. We analyzed germinated species traits ((Life_cycle: Perennial (P) vs. Annual (A); Palatability: High (HP), Medium (MP), Low (RP), Non-palatable (NP); and Germination area: Germination in grazed (G), protected (P) areas, or both (P+G)) along with response variables: frequencies across habitats (Above_protected, Above_grazed, SSB_protected, SSB_grazed). Redundancy analysis was then applied to evaluate these relationships. The results of this analysis show that the two main principal axes (PC1 and PC2) explain together 98.65% of the total variance (**Figure 8**). PC1 alone explains 74.79% of the variance, giving a significant gradient to understand the distribution of the germinated species. As explanatory variables, “Life cycle” is most influential compared to “palatability” and “germination area” indicating a lesser influence on the germinated species distribution. Life cycle arrow is oriented towards the left side of the PC1 suggesting a primary association with the species location. Conversely, palatability and germination area are oriented towards the right side of the PC1. As a response, frequencies (Above grazed and Above protected) are found with high positive loadings and strongly correlated in the positive side of PC1, while SSB_grazed and SSB_protected were in the negative side, correlating strongly with life cycle. The second RDA axis (PC2) showed high positive loading of SSB_grazed and lesser loading of SSB_protected. Based on the species distribution and correlations observed in this biplot, it appears that the group of species that are commonly present in the AGV cover in both protected and grazed areas are mostly perennials, having medium to high palatability with low germination frequencies either in protected or grazed areas. This group of species is mainly represented by *H. kahiricum*, *H. lippii*, *P. albicans*, and *A. uniflorum* (high frequencies in aboveground and very low germination frequencies). On the other side, the species frequently germinated, especially in SSB in grazed areas and less in SSB in protected areas, were mostly ephemerals that are of low palatability. This group is mainly represented by *I. spicata*, *F. germanica*, and *P. arabica* (low frequencies in aboveground and high germination frequencies). To this group of species is added *M. truncatula* with high germination frequencies only in the SSB of grazed areas. Species appearing in the lower right of this biplot were very frequent in all aboveground plots with low germination frequencies in soils from both protected and grazed areas. This group counted mainly *S. lagascae, M. longipetala, H. bicontorta, A. corrugatus* and *A. articulata*. On the lower-left side, clustered species were commonly registered at very low frequencies in both AGV and SSB germinated seedlings. They are mainly *H. spinosissimum*, *L. glomerata, L. nudicaulis, P. coronopus, S. glaucus* and *M. cerviana*. The intermediate left side of this RDA biplot shows a group of species dominated by annuals frequenting more grazed areas (AGV and SSB), which are *C. dichotoma, L. halophilus, B. madritensis*, and *D. sahariensis*, accompanied by a perennial *F. glutinosa*. It appears that protection favors the dominance of perennials with high and medium palatability in the aboveground, showing low germination frequencies from the SSB. Conversely, grazing favors the germination of annuals with low frequencies in the aboveground, likely due to competitive exclusion or grazing pressure of the most palatable ones. Some perennial species (e.g., *Stipa lagascae*) were frequent in AGV but had low SSB presence, suggesting reliance on vegetative persistence more than on seeds.

## 4 Discussion

Understanding the dynamics of pastoral ecosystems requires consideration of the multiple factors that influence interactions among their components. Ecological succession, which follows either a disturbance or the cessation of one, involves the gradual replacement of biological communities until a new state of equilibrium is established. Among the key factors shaping this process are climatic conditions and human activities. In the present study, we aim to evaluate vegetation dynamics—one of the major components of pastoral ecosystems—following a history of destructive disturbance (specifically, overgrazing) and the implementation of partial protection measures (protected areas). Particular attention is given to soil seed banks and their role in determining the composition and trajectory of successive vegetation communities.

Our main results confirmed that vegetation cover decreases under continuous grazing in arid areas, often accompanied by the formation of aeolian deposits and the appearance of stony surfaces, both indicating advanced soil degradation. Similar observations have been reported by Zhou et al. (2010) and Donovan and Monaghan (2021), who found that uncontrolled grazing accelerates soil instability and fertility loss. Under long-term protection, vegetation cover is higher and characterized by greater litter accumulation and well-developed soil crusts. The significant increases in above-ground vegetation (AGV), plant density, and species richness under protection involve both perennial and annual species, highlighting the negative impact of uncontrolled grazing on vegetation and soil surface structures. While several studies have documented positive vegetation dynamics under short-term protection (Tarhouni et al., 2014; Ebrahimi et al., 2016), the effects of long-term protection remain debated. Some authors (Wu et al., 2011; Ouled Belgacem et al., 2019; Chibani et al., 2022) have reported declines in biodiversity and ecosystem function, along with increased dominance of woody species (Su et al., 2015). More recently, Bartley et al. (2023) demonstrated that regenerative grazing can be a more profitable and sustainable alternative to other restoration practices, leading to notable improvements in ecosystem conditions.

Comparing floristic richness between the protected and grazed areas, the calculated similarity index indicates a moderate to high similarity (SCSI = 0.77). This suggests that important biodiversity and species richness are maintained even under overgrazing conditions, largely due to the dominance of unpalatable and/or grazing-resistant species. These findings are consistent with those reported by Tarhouni et al. (2017) and Msadek et al. (2021). Perennial species generally dominate the floristic composition across the study area, with Mediterranean and Saharan chorotypes remaining the most prevalent, while species of tropical origin are limited. Despite the pressures exerted on plant cover in recent decades, particularly due to climate change, the distribution of phytogeographical origins appears stable. This pattern is consistent with the observations of Le Houérou (1995) Such stability supports the conclusion of Chaieb et al. (1990), who reported a relative regression in the tropicality of the flora and an increase in its Mediterraneanity. This trend is especially evident among perennial grasses: for example, *Cenchrus ciliaris*, *Hyparrhenia hirta*, and *Stipagrostis ciliata* were recorded in the above-ground vegetation (AGV) of protected areas but were absent or present at very low frequencies in grazed sites. Conversely, Mediterranean perennial grasses such as *Stipa lagascae* and *Stipagrostis pungens* were found in the AGV of protected areas and were more frequent in grazed areas, although *Stipa lagascae* showed low seedling emergence in the soil seed bank (SSB) of both protected and grazed sites.

After two germination tests conducted on soil samples collected from two depths (0–3 cm and 3–6 cm) in both protected and grazed areas, our results showed the germination of only 30 species (38%) out of the 80 species recorded aboveground. Annual species dominated the germinated cohort, representing 63% of the total germinated species. In contrast to the aboveground vegetation composition, the germinated species showed higher annual density and richness in grazed areas, whereas no significant differences were observed for perennials across both tested depths.

Our findings are consistent with those of Miaojun et al. (2018), who reported that annuals contribute relatively more to soil seed banks (SSB) than perennials. Similarly, Huang et al. (2022) found that grazing exclusion enhances vegetation cover but reduces SSB density and diversity, leading to discrepancies between the soil seed bank and aboveground vegetation in alpine grasslands. In line with this, Chu et al. (2019) observed that increasing grazing intensity promotes greater species density and richness within the SSB. Furthermore, several studies (e.g., Dos Santos et al., 2018) suggest that seed banks in disturbed areas tend to contain more transient species—those with short life cycles, little or no dormancy, and high seedling recruitment potential—as well as a higher proportion of exotic species.

The germinated species belonged mainly to the *Fabaceae*, *Asteraceae*, *Poaceae*, and *Plantaginaceae* families, of which 67% were moderately to highly palatable. Similar findings were reported by Tekle and Bekele (2000), who observed that herbs constituted most emerged seedlings across various vegetation types (forests, shrublands, and grasslands) and degraded areas in Ethiopia, with four dominant families—*Asteraceae*, *Caryophyllaceae*, *Fabaceae*, and *Poaceae*—accounting for 62% of the total seedlings. Likewise, Wang et al. (2020) found that on the Loess Plateau in northern China, the *Asteraceae*, *Poaceae*, *Fabaceae*, and *Rosaceae* families comprised most of the emerged species from the soil seed bank. Comparable floral compositions were recorded in the dry tropical peri-urban ecosystems of the Bulandshahr–Khurja region near Delhi, where *Poaceae*, *Fabaceae*, and *Asteraceae* predominated (Narayan and Agrawal, 2015). In Saudi Arabia, Al-Huqail et al. (2025) similarly identified *Asteraceae*, *Poaceae*, *Boraginaceae*, and *Fabaceae* as major families, dominated by annual species. The widespread presence of *Asteraceae* in soil seed banks has been attributed to their high seed production and favorable morphological traits, such as small size, winged shape, and effective dispersal mechanisms (Hadinezhad et al., 2021). These authors also noted that forbs are more abundant than shrubs and trees in soil seed banks, even in areas where shrubs dominate above-ground vegetation, particularly in undisturbed sites.

Our findings indicate that the similarity between the above-ground vegetation (AGV) and the germinated soil seed bank (SSB) was higher in the grazed area than in the protected area. This suggests that long-term protection reduces seed input to the soil, thereby favoring the persistence of dormant seeds from perennial species. Although annual species were also present and emerged from the SSB in the protected area, their densities and frequencies were lower than those in grazed areas, reflecting the favorable conditions for seedling establishment provided by shrubs. According to Narayan and Agrawal (2015), annuals, characterized by short life spans and high fecundity, can rapidly colonize disturbed spaces. Studies conducted in Saudi Arabia revealed that significant differences between SSBs and AGV are largely due to the dominance of annuals and the presence of a substantial proportion of persistent seeds that remain dormant as a survival strategy under harsh climatic conditions (Al-Huqail et al., 2025). Similarly, Erfanzadeh et al. (2020) demonstrated that the composition of the SSB is influenced by the presence and type of shrubs, which create favorable microsites for seed trapping and germination.

Long-term grazing exclusion can reduce ecosystem resilience by promoting vegetation senescence and depleting the soil seed bank (SSB). Plant senescence under such conditions is often driven by reduced disturbance regimes, competitive exclusion, and altered nutrient cycling (e.g., Cabin et al., 2000; Maccherini and Santi, 2012). Natural disturbances—such as grazing, flooding, or fire—maintain vegetative vigor and diversity, whereas prolonged protection suppresses these processes. Over time, dominant species may outcompete others, reducing diversity and limiting regeneration, while biomass accumulation can disrupt nutrient cycling and lower soil fertility. Senescence further diminishes the SSB through reduced seed input, seed predation, absence of germination cues, and limited seed longevity. This is particularly pronounced in Mediterranean and arid ecosystems, where shrub seed banks are transient or short-term persistent and degrade without regular regeneration (Parker and Kelly, 1989).

This phenomenon was then reported in the gypsum soils, characteristic of southern Tunisia, hindering above-ground vegetation (AGV) dynamics and reducing the soil seed bank (SSB) through decreased seed storage. This process promotes lignification and favors the dominance of competitive species at the expense of ruderal and stress-tolerant species. Soil crust formation, which limits water infiltration and seed burial (Laker and Nortjé, 2019), may be considered a contributing factor to the decline of the soil seed bank (SSB) in long-term protected sites. Conversely, seeds dispersed outside these areas are more likely to become buried, either directly by wind deposition or indirectly through animal trampling. In general, prolonged grazing exclusion induces several changes in soil surface conditions, including soil crust formation, which reduces hydraulic functioning (Jeddi and Chaieb, 2010), diminished seed input into the soil, and litter accumulation that inhibits seed germination (Letts et al., 2015). Moreover, seeds remaining on the soil surface are increasingly exposed to predation (Dos Santos et al., 2013, Dos Santos et al., 2016).

The observed increase in similarity between AGV and SSB under grazing conditions can be attributed to the dominance of disturbance-tolerant species, which results in a greater proportion of transient seeds contributing directly to the visible plant community. Similar findings were reported by Dölle and Schmidt (2009), who concluded that SSBs in highly disturbed habitats are primarily composed of early successional species, and that the similarity between AGV and SSB is highest in such environments compared to less disturbed ones.

The redundancy analysis (RDA) conducted on all factors influencing the distribution frequency of germinated species in protected versus grazed areas revealed that the plant life cycle has a stronger influence than either palatability or location. This indicates that perennials dominate the aboveground vegetation (AGV), while annuals prevail in the soil seed bank (SSB), particularly under grazing pressure. Under arid conditions combined with overgrazing, the physiological and adaptive traits of plant species appear to play a decisive role in determining the successional patterns of plant communities (Dos Santos et al., 2013, Dos Santos et al., 2016).

The soil seed bank (SSB) plays a crucial role in shaping early successional stages of ecosystems. Despite the dominance of annual species in SSB, particularly in grazed areas, the phenomenon of therophytization persists under degradation pressures. Our findings align with those of Ouled Belgacem et al. (2013a); Hamida (2024), and Nora et al. (2025), which report the marked dominance of therophytes, primarily driven by harsh climatic conditions and overgrazing, especially in arid and desert regions. Similar patterns have been documented in southern Mediterranean areas, notably North Africa, including forest ecosystems. Barbero et al. (1990) reported the progressive replacement of trees and shrubs by therophytes, indicating forest steppization and therophytization under severe degradation.

In protected areas, palatable perennial species such as *Helianthemum* spp. and *P. albicans* dominate the AGV. However, their low frequency in the SSB suggests that their persistence relies mainly on vegetative propagation rather than seed reproduction. Conversely, grazing shifts the AGV composition toward less palatable annual species, likely due to competition and grazing pressure on palatable plants, while promoting the formation of a frequent and abundant seed bank consisting of species such as *Ifloga spicata*, *Schismus barbatus*, and *Filago germanica*.

Despite the general scarcity of perennial species in the SSB, possibly resulting from dormancy mechanisms and other physiological characteristics of persistent seeds, a notable frequency of certain palatable perennials, known for their resistance to overgrazing and abiotic stress, was recorded in grazed areas and reflected in the AGV composition. These species include *Argyrolobium uniflorum* (very frequent), *Plantago albicans*, *Helianthemum kahiricum*, *Helianthemum* sp., and *Stipa lagascae* (moderately frequent). The high abundance of these species in the disturbed area can be attributed to their resistance to grazing and their sensitivity to prolonged protection (Ouled Belgacem et al., 2013b). This observation is particularly relevant for identifying promising species for the rehabilitation of degraded rangelands in arid and semi-arid regions. Nevertheless, further studies are needed to build upon these findings.

We conclude that long-term protection promotes the persistence of palatable perennial plants in the AGV, whereas grazing favors the development of the SSB. This dynamic suggests that both the exclusion of grazing in protected areas and overgrazing in continuously grazed sites may generate recovery potential if grazing intensity is modified. Similar findings were reported by Dölle and Schmidt (2009), who observed that the regeneration capacity of vegetation from the SSB decreases markedly with increasing abandonment periods and/or decreasing disturbance intensity, while similarity between AGV and SSB increases with disturbance intensity. Likewise, Chu et al. (2019) found that the highest seed density occurred under light grazing, emphasizing that controlled grazing enhances SSB storage and plays a key role in vegetation dynamics.

Several studies have shown that the relationship between AGV and SSB is generally positive in the short term but becomes negative under long-term grazing exclusion (Huang et al., 2022; Kushbokov et al., 2025). Although short-term protection may have a positive effect on SSB during early successional stages, this effect should be interpreted with caution in later stages, particularly when the SSB is characterized by limited seed dispersal and low persistence (Wang et al., 2020). These factors should be carefully considered in future habitat restoration projects. Monitoring seed bank dynamics should be integrated into protected area management, and active management that mimics natural disturbances may be necessary.

Overall, these findings highlight the negative impacts of both continuous grazing and long-term protection on vegetation dynamics and AGV–SSB relationships. Therefore, regenerative (controlled) grazing is recommended as a balanced management approach that maintains ecosystem functions while preserving the positive roles of herbivores in aboveground and belowground vegetation dynamics.

## 5 Conclusion

This study demonstrates that grazing intensity and protection duration play decisive roles in shaping vegetation dynamics and soil seed bank (SSB) composition in arid pastoral ecosystems. Continuous grazing leads to a marked decline in vegetation cover, soil stability, and perennial plant abundance, whereas long-term protection favors the persistence of palatable perennial species and enhances aboveground vegetation (AGV) structure. However, prolonged exclusion also reduces seed input into the soil, resulting in a diminished and less diverse SSB.

Conversely, grazed areas maintain higher SSB density and diversity, dominated by annual and disturbance-tolerant species, which promote rapid colonization following disturbance. The higher similarity between AGV and SSB in grazed sites reflects the dominance of transient, short-lived species adapted to recurring stress. In contrast, the lower AGV–SSB similarity in protected sites indicates a decoupling between above- and belowground vegetation, driven by reduced disturbance and seed turnover.

Overall, both continuous grazing and prolonged exclusion negatively affect vegetation regeneration and ecosystem resilience. The results highlight that controlled or regenerative grazing, which balances disturbance and recovery by ensuring seed production and their entry into the soil, represents the most effective strategy for sustaining vegetation productivity, biodiversity, and soil functionality in arid and semi-arid rangelands. Additionally, given its confirmed importance, monitoring of seed bank status (viable seeds of key forage species) should become part of rangeland health assessment. Future management should therefore integrate moderate grazing regimes to maintain active seed banks and ensure the long-term ecological stability of pastoral ecosystems.

## Data Availability

The original contributions presented in the study are included in the article/supplementary material. Further inquiries can be directed to the corresponding author.

## References

[B1] Al-Huqail A. A. Al-Harbi H. F. Alowaifeer A. M. El-Sheikh M. A. Assaeed A. M. Alsaleem T. S. . (2025). Correlation between aboveground vegetation composition and soil seed bank of Raudhat desert habitat: a case study of Raudhat Alkhafs, Saudi Arabia. BMC Plant Biol. 25, 136. doi: 10.1186/s12870-025-06162-0. PMID: 39893391 PMC11786372

[B2] Barbero M. Bonin G. Loisel R. Quézel P. (1990). Changes and disturbances of forest ecosystems caused by human activities in the western part of the Mediterranean basin. Vegetatio 87, 151–173. doi: 10.1007/BF00042952. PMID: 41933263

[B3] Bartley R. Abbott B. N. Ghahramani A. Ali A. Kerr R. Roth C. H. . (2023). Do regenerative grazing management practices improve vegetation and soil health in grazed rangelands? Preliminary insights from a space-for-time study in the Great Barrier Reef catchments, Australia. Rangeland J. 44, 221–246. doi: 10.1071/RJ22047. PMID: 41161682

[B4] Benech-Arnold R. L. Sánchez R. A. Forcella F. Kruk B. C. Ghersa C. M. (2000). Environmental control of dormancy in weed seed banks in soil. Field Crops Res. 67, 105–122. doi: 10.1016/S0378-4290(00)00087-3. PMID: 41334505

[B5] Cabin R. J. Weller S. G. Lorence D. H. Flynn T. W. Sakai A. K. Sandquist D. . (2000). Effects of long‐term ungulate exclusion and recent alien species control on the preservation and restoration of a Hawaiian tropical dry forest. Conserv. Biol. 14, 439–453. doi: 10.1046/j.1523-1739.2000.99006.x. PMID: 41717205

[B6] Chaieb M. Floret C. Le Floc’h É. Pontanier R. (1990). Les graminées pérennes, un recours pour la réhabilitation des terres de parcours dégradés en zone aride tunisienne? Ecol. Mediterr. 16, 415–425.

[B7] Chibani R. Tlili A. Salem F. B. Louhaichi M. Ouled Belgacem A. Neffati M. (2022). Assessment of long-term protection on the aboveground biomass and organic carbon content using two non-destructive techniques: case of the Sidi Toui National Park in southern Tunisia. Afr J. Range For. Sci. 39, 281–291. doi: 10.2989/10220119.2021.1928752. PMID: 33685377

[B8] Chu H. Zhang C. Dong Q. Shang Z. Degen A. A. Yang X. . (2019). The effect of grazing intensity and season on the soil seed bank and its relation with above-ground vegetation on the alpine steppe. Agric. Ecosyst. Environ. 285, 106622. doi: 10.1016/j.agee.2019.106622. PMID: 41936479

[B9] Daget P. Poissonnet J. (1971). Principes d’une technique d’analyse quantitative de la végétation des formations herbacées. Doc. CEPE-CNRS 56, 85–100.

[B10] Dölle M. Schmidt W. (2009). The relationship between soil seed bank, above‐ground vegetation and disturbance intensity on old‐field successional permanent plots. Appl. Veg. Sci. 12, 415–428. doi: 10.1111/j.1654-109X.2009.01036.x. PMID: 41940437

[B11] Dong Q. Zhao X. Ma Y. Shi J. Wang Y. Li S. . (2012). Influence of grazing on biomass, growth ratio and compensatory effect of different plant groups in Kobresia parva meadow. Shengtai Xuebao / Acta Ecol. Sin. 32, 2640–2650. doi: 10.5846/stxb201103280398

[B12] Donovan M. Monaghan R. (2021). Impacts of grazing on ground cover, soil physical properties and soil loss via surface erosion: A novel geospatial modelling approach. J. Environ. Manage. 287, 112206. doi: 10.1016/j.jenvman.2021.112206. PMID: 33721762

[B13] Dos Santos D. M. da Silva K. A. de Albuquerque U. P. dos Santos J. M. F. F. Lopes C. G. R. de Lima Araújo E. (2013). Can spatial variation and inter-annual variation in precipitation explain the seed density and species richness of the germinable soil seed bank in a tropical dry forest in north-eastern Brazil? Flora 208, 445–452. doi: 10.1016/J.FLORA.2013.07.006. PMID: 41936479

[B14] Dos Santos D. M. Da Silva K. A. Dos Santos J. M. F. F. de Araújo E. L. (2018). Soil seed bank and its importance in the natural regeneration of degraded areas. Ethnobiol Conserv. 7, 5. doi: 10.15451/ec2018-03-07.05-1-7

[B15] Dos Santos D. M. dos Santos J. M. F. F. da Silva K. A. de Araújo V. K. R. de Lima Araújo E. (2016). Composition, species richness, and density of the germinable seed bank over 4 years in young and mature forests in Brazilian semiarid regions. J. Arid Environ. 129, 93–101. doi: 10.1016/j.jaridenv.2016.02.012. PMID: 41936479

[B16] Ebrahimi M. Khosravi H. Rigi M. (2016). Short-term grazing exclusion from heavy livestock rangelands affects vegetation cover and soil properties in natural ecosystems of southeastern Iran. Ecol. Eng 95, 10–18. doi: 10.1016/j.ecoleng.2016.06.069. PMID: 41936479

[B17] Erfanzadeh R. Shayesteh Palaye A. A. Ghelichnia H. (2020). Shrub effects on germinable soil seed bank in overgrazed rangelands. Plant Ecol. Divers. 13, 199–208. doi: 10.1080/17550874.2020.1718233. PMID: 41909888

[B18] Gamoun M. Ouled Belgacem A. Louhaichi M. (2018). Diversity of desert rangelands of Tunisia. Plant Divers. 40, 217–225. doi: 10.1016/j.pld.2018.06.004. PMID: 30740567 PMC6224667

[B19] Guo Q. Rundel P. W. Goodall D. W. (1998). Horizontal and vertical distribution of desert seed banks: patterns, causes, and implications. J. Arid Environ. 38, 465–478. doi: 10.1006/jare.1997.0353. PMID: 39885891

[B20] Hadinezhad M. Erfanzadeh R. Ghelichnia H. (2021). Soil seed bank characteristics in relation to different shrub species in semiarid regions. Land Degrad Dev. 32, 2025–2036. doi: 10.1002/ldr.3856. PMID: 41925065

[B21] Hamida M. (2024). “ The role of pre-desert vegetation in the rehabilitation of degraded soil,” in Vegetation dynamics - ecosystem management, conservation, and protection (London, UK: IntechOpen – London). doi: 10.5772/intechopen.1006659

[B22] Hu A. Zhang J. Chen X. Chang S. Hou F. (2019). Winter grazing and rainfall synergistically affect soil seed bank in semiarid area. Rangel Ecol. Manage. 72, 160–167. doi: 10.1016/j.rama.2018.07.012. PMID: 41936479

[B23] Huang M. Sang C. Zhao J. Degen A. A. Chen X. Zhang T. . (2022). Grazing exclusion altered the pattern of the soil seed bank but not the aboveground vegetation along an altitudinal gradient in alpine grassland. Land Degrad Dev. 33, 3901–3913. doi: 10.1002/ldr.4432. PMID: 41925065

[B24] Jauffret S. Visser M. (2003). Assigning life-history traits to plant species to better qualify arid land degradation in Presaharian Tunisia. J. Arid Environ. 55, 1–28. doi: 10.1016/S0140-1963(02)00258-6. PMID: 41881759

[B25] Jeddi K. Chaieb M. (2010). Changes in soil properties and vegetation following livestock grazing exclusion in degraded arid environments of South Tunisia. Flora 205, 184–189. doi: 10.1016/j.flora.2009.03.002. PMID: 41936479

[B26] Kalamees R. Püssa K. Zobel K. Zobel M. (2012). Restoration potential of the persistent soil seed bank in successional calcareous (alvar) grasslands in Estonia. Appl. Veg. Sci. 15, 208–218. doi: 10.1111/j.1654-109X.2011.01169.x. PMID: 41940437

[B27] Kushbokov A. Deák B. Valkó O. (2025). Characteristics of soil seed bank in global drylands – A review. Arid Land Res. Manage. 39, 289–310. doi: 10.1080/15324982.2025.2467728. PMID: 41909888

[B28] Laker M. C. Nortjé G. P. (2019). Review of existing knowledge on soil crusting in South Africa. Adv. Agron. 155, 189–242. doi: 10.1016/bs.agron.2019.01.002. PMID: 41936479

[B29] Le Houérou H. N. (1995). Bioclimatologie et biogéographie des steppes arides du Nord de l’Afrique. Options Méditerr Serie B, 10, 396.

[B30] Le Houérou H. N. Ionesco T. (1987). Palatabilité des espèces végétales de la Tunisie steppique (indices spécifiques) (Rome: Projet FAO/TUN–71/525. FAO, Division Production et Protection des Plantes).

[B31] Letts B. Lamb E. G. Mischkolz J. M. Romo J. T. (2015). Litter accumulation drives grassland plant community composition and functional diversity via leaf traits. Plant Ecol. 216, 357–370. doi: 10.1002/ece3.5469. PMID: 31463017 PMC6706195

[B32] Li X. R. Jia X. H. Long L. Q. Zerbe S. (2005). Effects of biological soil crusts on seed bank, germination and establishment of two annual plant species in the Tengger Desert (N China). Plant Soil 277, 375–385. doi: 10.1007/s11104-005-8162-4. PMID: 41933263

[B33] Louhaichi M. Gamoun M. Ben Salem F. Ouled Belgacem A. (2021). Rangeland biodiversity and climate variability: Supporting the need for flexible grazing management. Sustainability 13, 7124. doi: 10.3390/su13137124. PMID: 41725453

[B34] Maccherini S. Santi E. (2012). Long-term experimental restoration in a calcareous grassland: Identifying the most effective restoration strategies. Biol. Conserv. 146, 123–135. doi: 10.1016/j.biocon.2011.11.032. PMID: 41936479

[B35] Miaojun M. Jeffrey W. Zhen M. Lipei W. Guozhen D. (2018). Grazing disturbance increases transient but decreases persistent soil seed bank. Ecol. Appl. 28, 1020–1031. doi: 10.1002/eap.1706. PMID: 29710415

[B36] Msadek J. Tlili A. Moumni M. Louhaichi M. Tarhouni M. (2021). Community diversity, functional traits and adaptation of Stipa tenacissima L. under different grazing regimes in a North African arid montane rangeland. Afr J. Range For. Sci. 38, 122–129. doi: 10.2989/10220119.2020.1845796. PMID: 33685377

[B37] Narayan R. Agrawal S. (2015). “ Changes in plant diversity along disturbance gradient in a dry tropical region, India,” in Biodiversity in tropical ecosystems (New Delhi - India: Today and Tomorrow’s Printers and Publishers), 81–100.

[B38] Nefzaoui A. Salem H. B. El Mourid M. (2011). “ Innovations in small ruminants feeding systems in arid Mediterranean areas,” in New trends for innovation in the Mediterranean animal production (Brill, Wageningen, The Netherlands: Wageningen Academic), 99–116. doi: 10.3920/9789086867264_016, PMID:

[B39] Nora S. Hassen B. Farida B. Warda C. Abdelghafour D. Haroun F. . (2025). Floristic diversity and botanical composition of steppic Stipa tenacissima (L.) rangelands in El-Bayadh, Algeria. Livest. Res. Rural Dev. 37 (2). Available online at: https://www.researchgate.net/publication/392526897_Floristic_diversity_and_botanical_composition_of_steppic_Stipa_tenacissima_L_rangelands_in_El-Bayadh_Algeria.

[B40] Mohamed Y.O.S. Neffati M. Henchi B. (2002). Effet du mode de gestion des phytocénoses sur leur dynamique en Tunisie présaharienne: cas du parc national de Sidi Toui et de ses environs. Science et changements planétaires/Sécheresse, 13 (3), 195–203.

[B41] Ouled Belgacem A. Al Kaabi N. Al Wawi H. Louhaichi M. (2013a). Effect of livestock grazing on plant cover and species diversity in desert rangelands: A case study of Musawar Al Ottoria in Qatar. Range Mgmt Agrofor 34, 88–92. doi: 10.1002/ldr.1103, PMID: 41531421

[B42] Ouled Belgacem A. Ben Salem F. Gamoun M. Chibani R. Louhaichi M. (2019). Revival of traditional best practices for rangeland restoration under climate change in the dry areas: A case study from Southern Tunisia. Int. J. Clim. Change Strateg Manage. 11, 643–659. doi: 10.1108/IJCCSM-02-2018-0019. PMID: 35579975

[B43] Ouled Belgacem A. Tarhouni M. Louhaichi M. (2013b). Effect of protection on plant community dynamics in the Mediterranean arid zone of southern Tunisia: a case study from Bou Hedma national park. Land Degrad Dev. 24, 57–62. doi: 10.1002/LDR.110. PMID: 41925065

[B44] Palacio R. G. Bisigato A. J. Bouza P. J. (2014). Soil erosion in three grazed plant communities in northeastern Patagonia. Land Degrad Dev. 25, 594–603. doi: 10.1002/ldr.2289. PMID: 41925065

[B45] Parker V. T. Kelly V. R. (1989). “ Seed banks in California chaparral and other Mediterranean climate shrublands,” in Ecology of soil seed banks (Academic Press (New York), USA: Academic Press), 231–255.

[B46] Shang Z. Yang S. Wang Y. Shi J. Ding L. Long R. (2016). Soil seed bank and its relation with above-ground vegetation along the degraded gradients of alpine meadow. Ecol. Eng 90, 268–277. doi: 10.1016/j.ecoleng.2016.01.067. PMID: 41936479

[B47] Shen B. Ma Q. Q. Cheng Y. X. Chang S. H. Li Y. Guo J. M. . (2018). Effect of grazing systems on soil seedbank: A case study of an alpine meadow in the eastern Qinghai-Tibet Plateau. Pratacultural Sci. 35, 791–799.

[B48] Shi Y. F. Shi S. H. Huang X. M. Jiang Y. S. Liu J. Zhao Y. . (2022). A global meta‐analysis of grazing effects on soil seed banks. Land Degrad Dev. 33, 1892–1900. doi: 10.1002/ldr.4271. PMID: 41925065

[B49] Snyman H. A. (2004). Soil seed bank evaluation and seedling establishment along a degradation gradient in a semi-arid rangeland. Afr J. Range For. Sci. 21, 37–47. doi: 10.2989/10220110409485832. PMID: 33685377

[B50] Su H. Liu W. Xu H. Wang Z. Zhang H. Hu H. . (2015). Long‐term livestock exclusion facilitates native woody plant encroachment in a sandy semiarid rangeland. Ecol. Evol. 5, 2445–2456. doi: 10.1002/ece3.1531. PMID: 26120433 PMC4475376

[B51] Tarhouni M. Ben Hmida W. Neffati M. (2017). Long-term changes in plant life forms as a consequence of grazing exclusion under arid climatic conditions. Land Degrad Dev. 28, 1199–1211. doi: 10.1002/ldr.2407. PMID: 41925065

[B52] Tarhouni M. Ben Salem F. Ouled Belgacem A. Neffati M. (2010). Acceptability of plant species along grazing gradients around watering points in Tunisian arid zone. Flora 205, 454–461. doi: 10.1016/j.flora.2009.12.020. PMID: 41936479

[B53] Tarhouni M. Ben Salem F. Ouled Belgacem A. Neffati M. (2014). Impact of livestock exclusion on Sidi Toui National park vegetation communities, Tunisia. Int. J. Biodivers., 1–7. doi: 10.1155/2014/620405

[B54] Tekle K. Bekele T. (2000). The role of soil seed banks in the rehabilitation of degraded hillslopes in Southern Wello, Ethiopia 1. Biotropica 32, 23–32. doi: 10.1111/j.1744-7429.2000.tb00444.x. PMID: 41940437

[B55] Wang N. He X. Zhao F. Wang D. Jiao J. (2020). Soil seed bank in different vegetation types in the Loess Plateau region and its role in vegetation restoration. Restor Ecol. 28, A5–A12. doi: 10.1111/rec.13169. PMID: 41940437

[B56] Wellstein C. Otte A. Waldhardt R. (2007). Seed bank diversity in mesic grasslands in relation to vegetation type, management and site conditions. J. Veg. Sci. 18 (2), 153–162. doi: 10.1111/j.1654-1103.2007.tb02527.x. PMID: 41940437

[B57] Wu G. L. Li W. Li X. P. Shi Z. H. (2011). Grazing as a mediator for maintenance of off- spring diversity: sexual and clonal recruitment in alpine grassland communities. Flora 206, 241–245. doi: 10.1016/j.flora.2010.05.005. PMID: 41936479

[B58] Zhou Z. Gan Z. Shangguan Z. Dong Z. (2010). Effects of grazing on soil physical properties and soil erodibility in semiarid grassland of the Northern Loess Plateau (China). Catena 82, 87–91. doi: 10.1016/j.catena.2010.05.005. PMID: 41936479

